# Oral lichen planus interactome reveals CXCR4 and CXCL12 as candidate therapeutic targets

**DOI:** 10.1038/s41598-020-62258-7

**Published:** 2020-03-25

**Authors:** César Rivera, Mariangela Fernanda Crisóstomo, Carolina Peña, Paulina González-Díaz, Wilfredo Alejandro González-Arriagada

**Affiliations:** 1 Oral Medicine Group, Department of Stomatology, Faculty of Health Sciences, Universidad de Talca, Talca, Chile; 2 School of Dentistry, Faculty of Health Sciences, Universidad de Talca, Talca, Chile; 3 Servicio de Anatomía Patológica, Hospital Carlos Van Buren, Valparaíso, Chile; 4 Centro de Investigación en Ciencias Odontológicas y Médicas, Facultad de Odontología, Universidad de Valparaíso, Valparaíso, Chile; 5 Hospital El Carmen Dr. Luis Valentín Ferrada, Maipú, Santiago, Chile

**Keywords:** Oral diseases, Diseases, Dental diseases

## Abstract

Today, we face difficulty in generating new hypotheses and understanding oral lichen planus due to the large amount of biomedical information available. In this research, we have used an integrated bioinformatics approach assimilating information from data mining, gene ontologies, protein–protein interaction and network analysis to predict candidate genes related to oral lichen planus. A detailed pathway analysis led us to propose two promising therapeutic targets: the stromal cell derived factor 1 (CXCL12) and the C-X-C type 4 chemokine receptor (CXCR4). We further validated our predictions and found that CXCR4 was upregulated in all oral lichen planus tissue samples. Our bioinformatics data cumulatively support the pathological role of chemokines and chemokine receptors in oral lichen planus. From a clinical perspective, we suggest a drug (plerixafor) and two therapeutic targets for future research.

## Introduction

Oral lichen planus (OLP) is a chronic immunologically mediated disease^1^. Histopathological features of OLP include hyperkeratosis, acanthosis, degeneration by liquefaction of the basal keratinocyte layer, and prominent lymphocyte infiltration at the connective tissue–epithelium interface^2^. OLP is not a homogeneous clinical entity, but a complex disease. A recent review proposes a new classification of this disease, taking into consideration a set of lesions with similar characteristics, including oral lichenoid contact lesions, oral lichenoid drug reactions, and lichen planus pemphigoides, among others^3^.The clinical findings vary from white reticular plaques to erythema, erosions, ulceration, and hyperkeratotic plaques in the oral mucosa^4^. Although reticular lesions are often asymptomatic, burning or pain often accompanies erosive/ulcerative manifestations of OLP. The severity of pain may vary from mild to severe and may even interfere with patients’ speech, feeding, and swallowing^4^. According to the WHO, this disease has a potential for cancerization^5^. This potential is presented in about 1% of patients^6^. Despite the efforts in biomedical research, the etiology of OLP is unknown, and its molecular basis is still unclear. This may explain why the treatment is palliative. For these reasons, exploration of the mechanisms that govern the disease’s pathophysiological process is needed.

A disease is rarely the result of an abnormality in a single gene, but it reflects disturbances in the complex intracellular and intercellular network that links tissue and organ systems^7^. Today, we face difficulty in generating new hypotheses and understanding OLP due to the large amount of biomedical information available. Encapsulating that information in an understandable way is one of the current challenges of network-based approaches to the study of human diseases^8^. The heterogeneity of pathologies, the difficulty of finding a gene or protein in a certain localization, and the cost involved in experimental studies have led to the development of several in silico approaches for the identification of genes and proteins associated with a disease^9^. These bioinformatic approaches address complexity by simplifying systems, summarizing them as interaction maps or interactomes, and containing components (nodes) and lines (interactions) between them^7^. To explore biological systems, analyses involve data mining^10^, protein–protein interactions^11^, gene ontology (GO)^12^, gene regulatory networks, and pathways for gene identification candidates^13^. Several publications have reported on the successful use of such techniques to prioritize disease-related genes and propose therapeutic targets^14–16^. However, these technologies have not been applied to the study of OLP.

In order to better understand the molecular basis of OLP and propose new therapeutic targets, we use data mining to obtain a large number of protein coding genes associated with this disease. Then, we describe the biological attributes of proteins and prioritize a small group of them using protein–protein interaction networks. With the most relevant proteins, we defined the OLP interactome, which allowed us to propose a new treatment for the disease and two therapeutic targets. Finally, we check our computational predictions by immunohistochemistry.

## Methods

### General design

**Figure 1:**
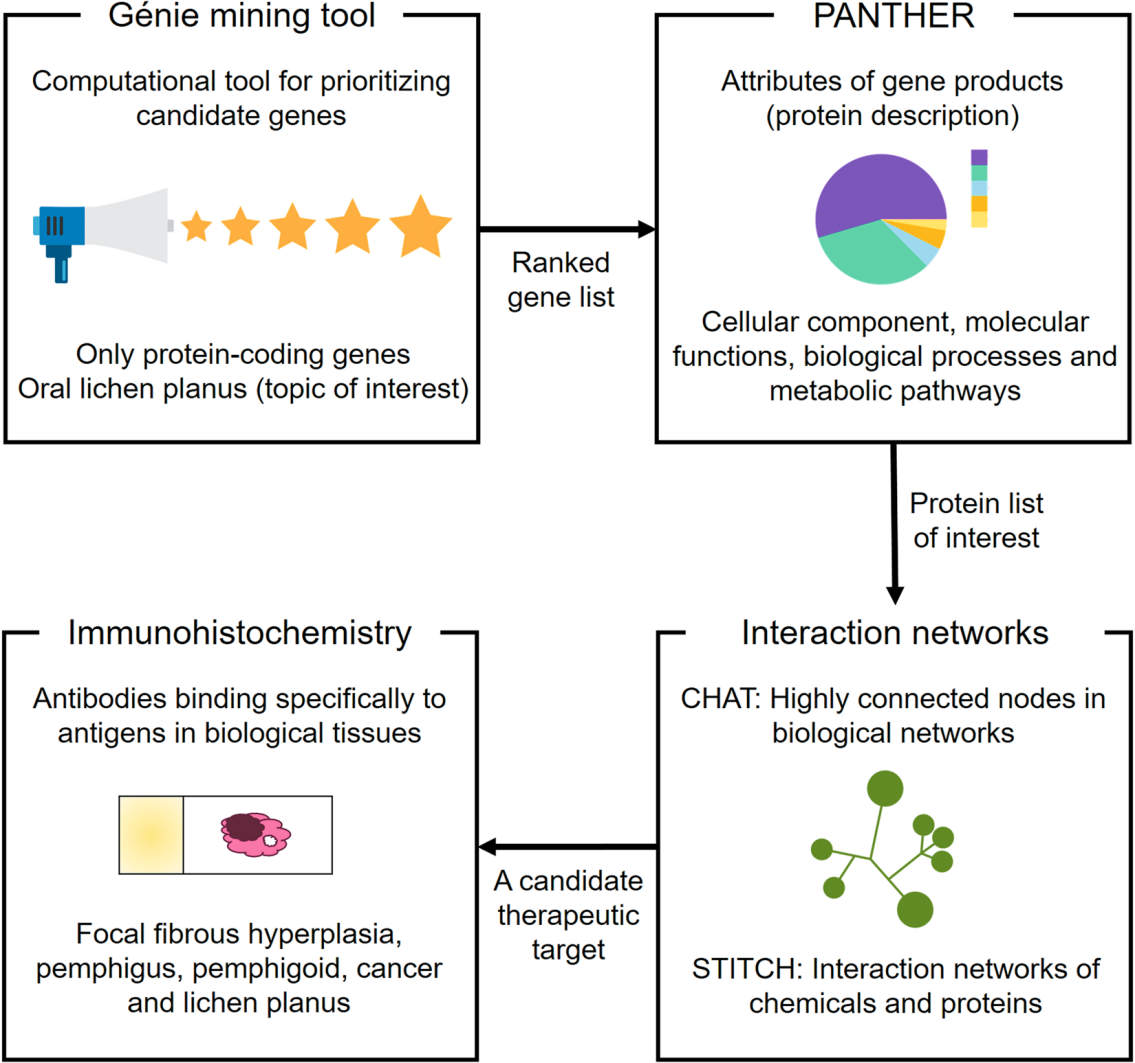
Experimental design. Initially, we used Génie web server to perform a ranking of protein-coding genes associated with oral lichen planus (OLP). Then, using PANTHER, we characterized them based on gene ontology (controlled vocabulary that describes the gene and the attributes of the gene product). The most important proteins of that set (which we call the OLP interactome) were prioritized by building interaction networks (CHAT). From them, we identified drug target proteins (STITCH). One of the proposed targets, a chemokine receptor, was evaluated (immunohistochemistry) in a series of cases of focal fibrous hyperplasia (a reactive hyperplastic lesion of the connective tissue in response to local irritation), head and neck cancer (malignant neoplasm), pemphigus and pemphigoid (blistering autoimmune diseases), and OLP.

Figure 1 provide a brief description of our workflow. All procedures were in accordance with the Helsinki Declaration^17^. The Ethics Committee of UTALCA (protocol #2017-02-CR) approved this research (10.5281/zenodo.3576206).

### Protein identification and classification

To identify genes encoding proteins related to OLP from a large volume of information, we use the Génie web tool^18^. This tool analyzes relationships between genes and biomedical topics in all abstracts available on MEDLINE/PubMed. The topic of interest was defined by the search (carried out on July 19, 2018): “lichen planus, oral” [MeSH] and “biomarkers” [MeSH] and (risk ratio [Title/Abstract] or relative risk [Title/Abstract] or odds ratio [Title/Abstract] or risk [Title/Abstract]) and (“humans” [MeSH Terms]). With these criteria, 23 abstracts were identified as a training set. In order to find only significant coding genes, we established as a cut-off p-value of <0.01 for abstracts and a false positive discovery rate (FDR) of <0.01 for genes. We use Fisher’s exact test to define the relationship between genes and search topics.

The gene list was subjected to a gene ontology analysis using the PANTHER representation test (http://www.pantherdb.org)^19^ version 13.1 to determine classifications based on biological processes, cellular components, molecular functions, and metabolic pathways. We select the coding genes from the most overrepresented classifications.

### Interactome construction

To build the OLP interactome, we use the Contextual Hub Analysis (CHAT) application of the Cytoscape program^20^ and the STITCH web tool (http://stitch.embl.de/)^21^. CHAT identifies the genes of greatest relevance within a list. For this, it builds a molecular network composed of (i) the list of genes (each gene is a node), (ii) the numerical values for each gene (for example, expression or belonging to a group), and (iii) a database with the genes that will establish interactions. The smallest p-values represent the centers of greatest activity within the biological network (hubs). We assign a numerical value of +3 (context attribute) to all coding genes included in the overrepresented PANTHER categories, and as a database for the interactions, we select IntAct^22^. To establish whether the resulting hubs corresponded to a biologically connected network, we used STITCH. These base reports known interactions as well as establishing predictions of interactions between proteins and chemicals. In STITCH, we choose to establish interactions with the maximum confidence level (0.9) using all available resources. We call the resulting network the “OLP interactome.”

### Immunohistochemistry verification

We retrospectively collected focal fibrous hyperplasias (*n*  =  10), head and neck cancers (n = 4), pemphigoid (n = 2), pemphigus (n = 2) and OLP lesions (*n*  =  12) in paraffin-embedded tissues stored in the UTALCA Biobank/Oral Pathology Laboratory (https://medicinaoral.pro/biobanco). The information of patients is provided in Supplementary Table 1 (10.5281/zenodo.3483255). For OLP diagnosis, we use both clinical and histopathologic criteria enumerated in the position paper by the American Academy of Oral and Maxillofacial Pathology^2^. Immunostaining of 3 μm histological sections was performed using EnVision FLEX target retrieval solution (High pH, Dako) according to our previously published protocols^23,24^. The primary antibody used was a CXCR4 antibody (1:1000 dilution, #PA3305, Invitrogen Inc., USA), used overnight at 4 °C. Two pathologists blinded to the clinical data provided a consensus opinion of staining patterns. We use oral cancer cases as positive controls. Also, evidence that CXCR4 antibody represent a specific staining can be consulted in our previous studies^23,24^.

## Results

### OLP proteins participate in inflammation mediated by chemokines and cytokines

With Génie, we obtained 872 statistically significant protein-coding genes extracted from 1,075,776 articles (Supplementary Dataset 1, 10.5281/zenodo.3483255). To discover patterns in that large volume of information, we describe the set using PANTHER. This tool allowed us to classify gene products (that is, proteins) into four categories: the cell zone in which they are found (cellular component), the molecular functions, biological processes, and metabolic pathways in which they participate. The visual interface offers pie charts, in which we click on the categories that incorporated a greater number of genes. This allowed us to “dive” to the root of each topic to find more specific information. Table 1 shows the most relevant processes, which are represented by 51 proteins. Considering the recognized role of immune mediation in OLP, the metabolic pathway corresponding to inflammation mediated by chemokines and cytokines stands out. The complete classification can be consulted in Supplementary Dataset 2 (10.5281/zenodo.3483255).

**Table 1:** Classification of genes associated with oral lichen planus.

Functional classification	Genes
Panther GO-Slim Cellular Component:Cytoplasm	TP73, GUCY2C, SOCS3, HSPA1B, AKR1A1, RRM2, GOLPH3, AKR1C3, NOS3, AR, MTRR, PDCD4, MTHFD1, SOCS1, NOS2, PIK3CA, HSPA1L, TP53, NOS1, IRS2, IRS1, MTHFR, MTR, MTHFD2, TP63, (Cytosol, GO:0005829)
Panther GO-Slim Biological Process: MAPK cascade	MAP2K4, KALRN, (JNK cascade, GO:0007254)
Panther GO-Slim Molecular Function: Cytokine receptor binding	GDF15, TGFB1, TGFB3, (Transforming growth factor beta receptor binding, GO:0005160)
Panther Pathway: Inflammation mediated by chemokine and cytokine signaling pathway	CXCL8, CCL11, CCL2, CXCL10, CCL18, CCL5, CCL21, CCL8, CCL22, CCL20, (Chemokine, P00856), CX3CR1, CCR6, CXCR1, CCR7, CCR3, CXCR2, CXCR4, CCR6, CCR1, CCR5, CCR2, (Chemokine receptor, P00854)

Genes significantly associated with oral lichen planus were analyzed in PANTHER. The most specific and relevant subcategories of each group are listed.

### OLP interactome reveals two promising therapeutic targets for plerixafor

**Figure 2:**
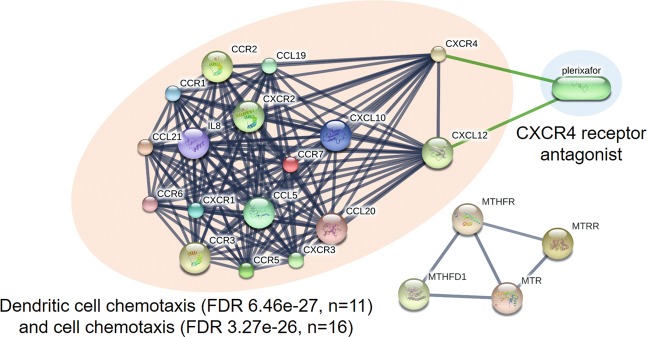
Interactome of oral lichen planus. Network with 21 proteins processed by STITCH and its pharmacological relationship with plerixafor. The clustering coefficient is 0.968. Thicker lines represent stronger associations. Protein–protein interactions are shown in gray, and chemical–protein interactions in green. FDR = false discovery rate. The original network can be consulted at http://stitch.embl.de/cgi/network.pl?taskId=L9CBnAWsnpeX.

For a better interpretation of the data in a biological context, we evaluate the proteins using the Cytoscape program and its CHAT application. CHAT identifies central nodes (proteins with many connections) that interact with more “contextual” nodes (i.e., 51 proteins obtained from the previous step). CHAT calls these nodes “contextual hubs.” These hubs have topological and functional relevance, since their elimination causes great damage in a network, so they are the best representatives of a biological system^25^. The application built an interaction network of 1,045 nodes using the IntAct database as a source (Supplementary Fig. 1, 10.5281/zenodo.3483255). From this network, we selected the most important hubs (17 significant p-values reported by CHAT, Supplementary Dataset 3, 10.5281/zenodo.3483255). Next, we identified the interactions between chemicals and proteins. Using STITCH, we identified a network composed of 21 proteins with a high clustering coefficient (0.97), which indicates that it is highly feasible that these proteins are a biologically interconnected community. We call that network the OLP interactome. In that network, two proteins interact with plerixafor: the stromal cell–derived factor 1 (CXCL12) and the C-X-C type 4 chemokine receptor (CXCR4) (Fig. 2).

### CXCR4 is overexpressed in connective tissue of OLP lesions

**Figure 3:**
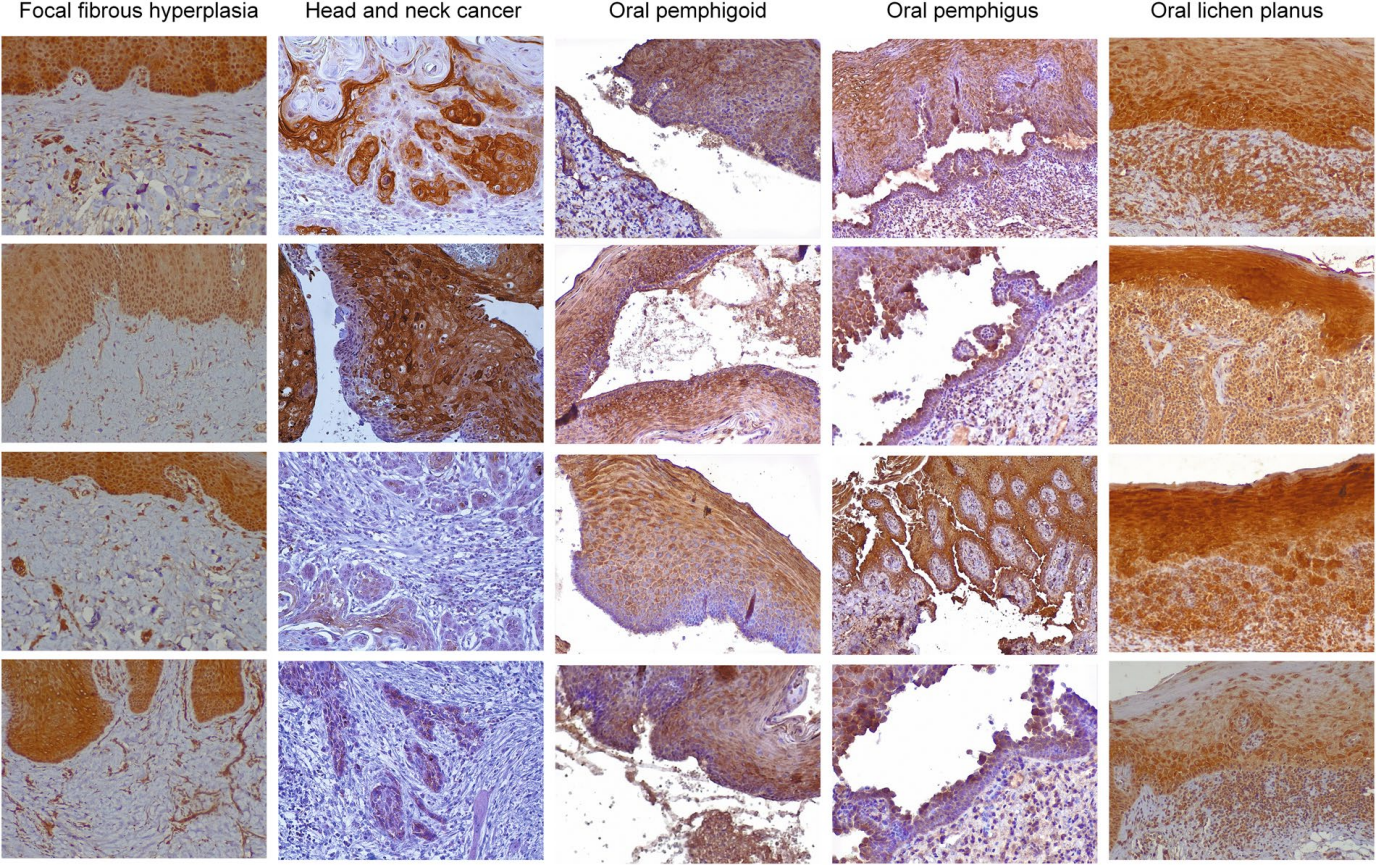
CXCR4 is overexpressed in the connective tissue of oral lichen planus. Representative microphotographs of CXCR4 receptor immunohistochemical staining in oral tissues (40× objective). Positive staining is shown in brown. Compared to the controls, oral lichen planus lesions show high intensity and reactivity to CXCR4 in areas of active inflammatory infiltrate. All images (n = 99) can be reviewed at, 10.5281/zenodo.3352836.

To confirm our bioinformatic predictions, we used immunohistochemistry. We detected higher expression of CXCR4 in OLPs than in other lesions (fibrous hyperplasia, cancer, pemphigoid and pemphigus, Fig. 3). Epithelial staining reactivity were positive in all samples. In the subepithelial connective tissue, the differences are remarkable. A high marking intensity for CXCR4 is present in all OLP cases. CXCR4 staining coincides with inflammatory areas. These results may indicate the presence of actively infiltrating immune cells, which are positive for this receptor.

## Discussion

The identification of genes involved in diseases is an important tool to reveal molecular mechanisms for disease development and for the establishment of new therapies^26^. Here, we have predicted and prioritized a group of proteins associated with OLP along with proposing two possible therapeutic targets for the disease, CXCL12 and its receptor, CXCR4.

Connecting genes and proteins with the diseases, in which they are involved, is the heart of molecular medicine^27^. Several applications have been developed to allow genes to be linked with complex diseases^26–31^. One of them is Génie, which has been previously used in the determination of risk genes for non–small cell lung cancer^32^. Using this tool, we obtained a set close to 1,000 genes coding for proteins related to OLP, among which chemokine and cytokine-mediated inflammation stand out. These findings are in accordance with the current evidence that define this disease focused on its chronic and immunological basis^33^.

With our prioritization analysis, we identify the oral lichen planus interactome, consisting of two clusters of 21 proteins: CXCL10, CXCL12, CCL5 (RANTES), CCL19, CCL20, CCL21, CXCR1, CXCR2, CXCR3, CXCR4, CCR1, CCR2, CCR3, CCR5, CCR6, CCR7, IL-8, MTHFR, MTR, MTRR, and MTHFD1. The main cluster includes chemokines and chemokine receptors participating in dendritic cell chemotaxis. In addition, these proinflammatory molecules are produced by cells primarily to recruit leukocytes at sites of infection or injury^34^. For example, CXCL10 and CXCL12 are chemoattractors of T lymphocytes and monocytes^35,36^; CCL5 of monocytes, memory T-helper lymphocytes, and eosinophils^37^; CCL19 of T and B lymphocytes^38^; and IL-8 of neutrophils, basophils, and T lymphocytes^39^. The fact that 17 lichen planus interactome proteins are chemokines or their receptors explains why the lymphocytic infiltrate in this disease is intense in the connective tissue, resembling a band^40^. The intensity of this infiltrate leads to overlying keratinization and degeneration due to liquefaction of the basal layer^41^, the latter directed by CD8 + auto-cytotoxic T-lymphocytes^1^. Our interactome suggests that cell-mediated histological features are triggered by a large flow of chemokines.

The switch that starts the mechanisms of oral lichen planus is still unknown. Revealing the molecules that constitute the major centers of activity or disturbance points in the OLP network can provide a chance to find new therapies. Surprisingly, using STITCH, we predict that plerixafor has two therapeutic targets, CXCR4 and CXCL12.

The CXCL12-CXCR4 axis regulates leukocyte chemotaxis in inflammatory conditions and autoimmune diseases. It has significantly been studied in numerous cancers and autoimmune diseases^42^. This axis modulates effects on cells in an autoimmunity context, which may be important for the development or severity of various diseases, including psoriasis, multiple sclerosis, rheumatoid arthritis, systemic lupus erythematosus, idiopathic inflammatory bowel diseases, and type 1 diabetes^43^. Recent experimental evidence shows that the CXCL12-CXCR4 axis participates in inappropriate retention of activated innate inflammatory cells at inflammatory sites. This is highly relevant for chronic diseases, such as chronic obstructive pulmonary disease and asthma^44^. The broad participation of the CXCL12-CXCR4 axis in several diseases justifies the recognition of antagonistic drugs.

Plerixafor, also known as AMD3100, was originally developed as a drug against human immunodeficiency virus and then characterized as a CXCR4 antagonist^45^. Plerixafor has proven to be useful for inhibiting the CXCL12-CXCR4axis in patients with leukemia^46^. Although there is no evidence of the usefulness of this drug in OLP, a biological basis supports this therapeutic use.

Previous evidence shows that the microdissected oral epithelium of OLP patients presents an increase in gene expression of 258% for CXCL12 and 629% for CXCR4^47^. In addition, our immunohistochemistry corroborated the predictions. We tested the expression of CXCR4 for two reasons: first, because our research group has previous studies with this protein^23,24^ and second, the immunohistochemistry method is poorly quantitative and can suffer from low sensitivity for detection of secreted proteins (for example, CXCL12)^48^.

We observed that CXCR4 is highly expressed in the connective tissue of OLP patients. It is known that CXCR4 and CXCL12 are expressed in dendritic cells (Langerhans cells)^49,50^. Since dendritic cells are highly activated in OLP, this may be an important event for disease development. Dendritic cells are the most potent antigen presenting cells for lymphocytes^51,52^. These cells present the antigen to memory T cells, the predominant phenotype in OLP^53^. It is known that CXCR4 is highly-expressed in resting T cells, including naïve and memory T cells, and is downregulated during T cell activation^54^. We believe that the presence of high amounts of CXCR4 may represent populations of dendritic cells, memory T cells, or a combination of both.

An initial event in the disease mechanism may involves the expression or presentation of the keratinocyte antigen (still unknown) to trigger the immune response^1^. Therefore, it is logical to think that an intervention that interrupts the triggering of lymphocytic infiltrate could ensure epithelial integrity. Although topical steroids are considered the first-line treatment for symptomatic oral lichen planus, there is no evidence to support the effectiveness of these drugs^55,56^, which we believe is an invitation to explore new options.

The initial search for our design does not distinguish between the clinical variants of OLP; however, our analyses are inspired by the erosive/ulcerative forms that are accompanied by painful symptomatology. Our results are limited to the performance of our applications in silico, and new studies are needed to provide experimental data for our analysis. In the future, we should test whether the high expression of CXCR4 can be verified using other techniques, such as PCR experiments (after tissue microdissection) or salivary ELISA.

In this investigation, bioinformatics data cumulatively support the pathological role of chemokines and chemokine receptors in OLP. From a clinical perspective, we suggest a drug and two therapeutic targets for future research. Additionally, we demonstrate that it is possible to comprehensively analyze a large volume of biomedical information in order to better understand the molecular complexity of the OLP, even confirming a bioinformatics prediction using immunohistochemistry. We believe in the need for future studies to verify and monitor – experimentally and clinically – the role of the proteins listed in this research to move towards more precise therapies.

## Supplementary information

Supplementary Table 1.

upplementary Dataset 1.

upplementary Dataset 2.

upplementary Dataset 3.

Supplementary Figure S1.

## Data Availability

All supplementary files are included in this article and are publicly available at: 10.5281/zenodo.3483255.
