# Supplementary material for: Oral lichen planus interactome reveals CXCR4 and CXCL12 as candidate therapeutic targets
Source: Sci Rep. 2020 Mar 25;10:5454. doi: 10.1038/s41598-020-62258-7 (PMC7096434; doi:10.1038/s41598-020-62258-7)
Supplement: Supplementary file 1 — Supplementary Table 1. [file 41598_2020_62258_MOESM1_ESM.docx]

**Supplementary table 1**. Clinicopathological characteristics of patients.

| **#** | **Cases** | **Diagnosis** | **Age** | **Sex** | **Location** | **Laterality** |
| --- | --- | --- | --- | --- | --- | --- |
| 1 | UV-17-10 | Focal fibrous hyperplasia | 55 | Woman | Jugal Mucosa | Left |
| 2 | UV-18-06 | Focal fibrous hyperplasia | 57 | Woman | Jugal Mucosa | Right |
| 3 | UV-18-13 | Focal fibrous hyperplasia | 53 | Woman | Jugal Mucosa | Left |
| 4 | UV-18-31 | Focal fibrous hyperplasia | 47 | Man | Upper gum | Left |
| 5 | UV-18-39 | Focal fibrous hyperplasia | 33 | Woman | Upper gum | Left |
| 6 | UV-18-41 | Focal fibrous hyperplasia | 47 | Woman | Jugal Mucosa | Right |
| 7 | UV-18-50 | Focal fibrous hyperplasia | 44 | Woman | Tongue dorsum | Central |
| 8 | UV-18-52 | Focal fibrous hyperplasia | 68 | Woman | Lower lip | Left |
| 9 | WG-18-08 | Focal fibrous hyperplasia | 64 | Man | Upper lip | Central |
| 10 | WG-18-17 | Focal fibrous hyperplasia | 60 | Woman | Jugal Mucosa | Left |
| 11 | UV-06-09 | Oral lichen planus | 50 | Woman | Upper gum | Left |
| 12 | UV-07-11 | Oral lichen planus | 21 | Man | Jugal Mucosa | Left |
| 13 | UV-09-26 | Oral lichen planus | 61 | Woman | Upper gum | Central |
| 14 | UV-14-26 | Oral lichen planus | 77 | Woman | Jugal Mucosa | Left |
| 15 | UV-15-15 | Oral lichen planus | 79 | Woman | Jugal Mucosa | Left |
| 16 | UV-15-29 | Oral lichen planus | 50 | Woman | Lateral border of tongue | Left |
| 17 | UV-15-50 | Oral lichen planus | 50 | Man | Lateral border of tongue | Right |
| 18 | UV-16-31 | Oral lichen planus | 62 | Man | Trigone | Right |
| 19 | UV-16-50 | Oral lichen planus | 53 | Man | Trigone | Left |
| 20 | UV-17-02 | Oral lichen planus | 26 | Man | Jugal Mucosa | Left |
| 21 | UV-17-63 | Oral lichen planus | 54 | Woman | Jugal Mucosa | Left |
| 22 | UV-18-14 | Oral lichen planus | 66 | Woman | Jugal Mucosa | Left |
| 23 | 3033-12 | Head and neck cancer | 64 | Woman | Tongue | - |
| 24 | 3466-14 | Head and neck cancer | 54 | Woman | Larynx | - |
| 25 | 4326-07 | Head and neck cancer | 68 | Man | Tongue | - |
| 26 | 5199-14 | Head and neck cancer | 36 | Woman | Tongue | - |
| 27 | UV-16-86 | Pemphigoid | 26 | Woman | Attached gum | NI |
| 28 | UV-16-70 | Pemphigoid | 58 | Man | Attached gum | NI |
| 29 | UV-16-56 | Pemphigus | 66 | Woman | Jugal Mucosa | NI |
| 30 | UV-15-46 | Pemphigus | 37 | Woman | Jugal Mucosa | NI |

For OLP diagnosis we use both clinical and histopathologic criteria enumerated in the position paper by the American Academy of Oral and Maxillofacial Pathology (1). NI, not informed.

**REFERENCE**

1. Cheng YS, Gould A, Kurago Z, Fantasia J, Muller S. Diagnosis of oral lichen planus: a position paper of the American Academy of Oral and Maxillofacial Pathology. Oral Surg Oral Med Oral Pathol Oral Radiol. 2016;122(3):332-54.
