# Supplementary material for: Oral lichen planus interactome reveals CXCR4 and CXCL12 as candidate therapeutic targets
Source: Sci Rep. 2020 Mar 25;10:5454. doi: 10.1038/s41598-020-62258-7 (PMC7096434; doi:10.1038/s41598-020-62258-7)

**Supplementary figure 1.** Interaction network using the Interactome database in CHAT/Cytoscape program. The main activity centers are represented by a gradient of color. The 17 main highly connected nodes (hubs) were are selected for further analysis with STITCH.


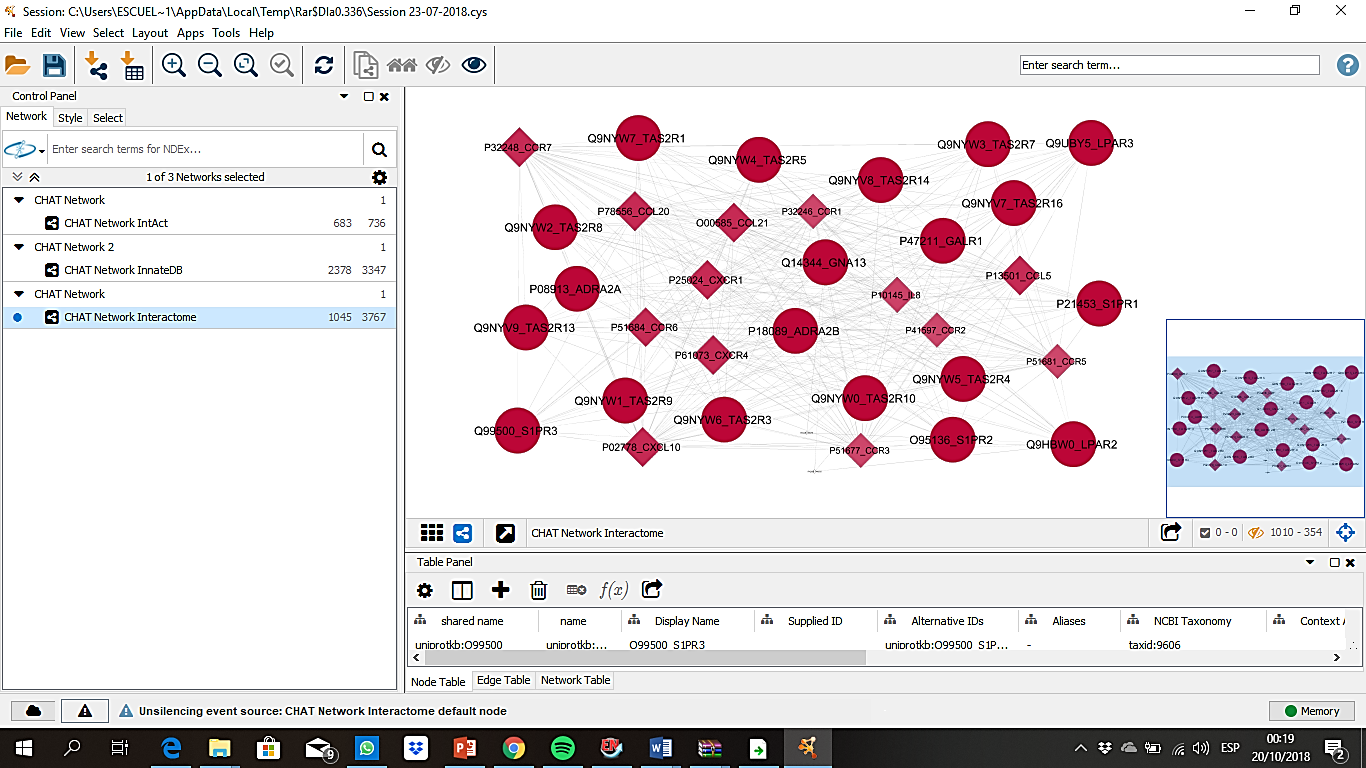

Supplement: Supplementary file 5 — Supplementary Figure S1. [file 41598_2020_62258_MOESM5_ESM.docx]
